# BcCFEM1, a CFEM Domain-Containing Protein with Putative GPI-Anchored Site, Is Involved in Pathogenicity, Conidial Production, and Stress Tolerance in *Botrytis cinerea*

**DOI:** 10.3389/fmicb.2017.01807

**Published:** 2017-09-20

**Authors:** Wenjun Zhu, Wei Wei, Yayun Wu, Yang Zhou, Fang Peng, Shaopeng Zhang, Ping Chen, Xiaowen Xu

**Affiliations:** ^1^College of Biology and Pharmaceutical Engineering, Wuhan Polytechnic University Wuhan, China; ^2^Institute for Interdisciplinary Research, Jianghan University Wuhan, China; ^3^State Key Laboratory of Agrobiotechnology and Ministry of Agriculture Key Laboratory of Plant Pathology, China Agricultural University Beijing, China

**Keywords:** *Botrytis cinerea*, *BcCFEM1*, GPI-anchored site, CFEM domain, virulence, stress tolerance

## Abstract

We experimentally isolated and characterized a CFEM protein with putative GPI-anchored site BcCFEM1 in *Botrytis cinerea*. BcCFEM1 contains a CFEM (common in several fungal extracellular membrane proteins) domain with the characteristic eight cysteine residues at N terminus, and a predicted GPI modification site at C terminus. *BcCFEM1* was significantly up-regulated during early stage of infection on bean leaves and induced chlorosis in *Nicotiana benthamiana* leaves using *Agrobacterium* infiltration method. Targeted deletion of *BcCFEM1* in *B. cinerea* affected virulence, conidial production and stress tolerance, but not growth rate, conidial germination, colony morphology, and sclerotial formation. However, over expression of *BcCFEM1* did not make any observable phenotype change. Therefore, our data suggested that BcCFEM1 contributes to virulence, conidial production, and stress tolerance. These findings further enhance our understanding on the sophisticated pathogenicity of *B. cinerea* beyond necrotrophic stage, highlighting the importance of CFEM protein to *B. cinerea* and other broad-host-range necrotrophic pathogens.

## Introduction

The ascomycetous fungus *Botrytis cinerea* is an economically important and destructive necrotrophic fungal pathogen with the capability of causing gray mold disease on more than 200 plant species (Williamson et al., 2007). *B. cinerea* is also responsible for leading to serious economic losses each year in harvested fruits and vegetables during storage stage (van Kan, 2006; Williamson et al., 2007), and has been ranked as the second most important plant pathogenic fungus (Dean et al., 2012).

According to lifestyles, plant pathogens are distinguished as biotrophic, necrotrophic, and hemibiotrophic pathogens. Biotrophic pathogens colonize and acquire nutrition directly from living plant cells and tissues. Necrotrophic pathogens kill host plant cells prior to or during colonization, and feed on dead plant tissue. Hemibiotrophic pathogens initially establish an biotrophic phase with living cells and subsequently switch into the necrotrophic phase and kill plant cells for nutrient acquisition.

The broad-host-range necrotrophs, such as *B. cinerea* and *Sclerotinia sclerotiorum*, were believed to be typical necrotrophic fungi, originally viewed as aggressive pathogens secreting multiple of toxins, plant cell-wall degrading enzymes and proteinases to rapidly destroy and kill host cells and tissues (Riou et al., 1992; Choquer et al., 2007). But now, they are believed to adopt more sophisticated and comprehensive strategies for infection than previously considered before killing the host plants (Shlezinger et al., 2011, 2016; Williams et al., 2011; Kabbage et al., 2013, 2015; van Kan et al., 2014). However, compared with biotrophic and hemibiotrophic pathogens, the underlying interaction mechanisms between plants and broad-host-range necrotrophic pathogens remain incompletely understood, especially in the early infection stage.

Glycosylphosphatidylinositol (GPI)-anchored proteins, which anchored to the outer layer of the plasma membrane through a C-terminal GPI anchor, are essential for growth, viability, morphogenesis, signaling transmission, reproduction, biofilm formation, surface adhesion, and disease pathogenesis in all eukaryotic cells (Eisenhaber et al., 2001). In animal cells, GPI-anchored proteins were involved in development of wing in *Drosophila* (Kreuger et al., 2004). Mutation of GPI synthesis caused lethal phenotype of embryo in mice (Kawagoe et al., 1996). Deletion of mice *PGAP1* gene, which encodes inositol deacylase, caused otocephaly and male infertility (Ueda et al., 2007). In plants, GPI-anchored proteins contributed importantly to the regulation of cell expansion and cell wall biosynthesis (Brady et al., 2007), embryo lethality and male fertility (Dai et al., 2014), pollen tube-female gametophyte interaction and growth (Capron et al., 2008; Li et al., 2013; Duan et al., 2014), and fertilization and early seed development (Tsukamoto et al., 2010). In *Saccharomyces cerevisiae*, mutation of GPI10 cause cell death in yeast (Sütterlin et al., 1998). Multiple GPI-modified wall proteins of yeast were important to osmotic stability and mortality (Teparić et al., 2004). GPI-anchored membrane proteins DCW1 and DFG5 are essential for cell growth and cell wall biogenesis in *S. cerevisiae*, double deletion caused fragile cell wall and is lethal (Kitagaki et al., 2002, 2004). Nevertheless, the specific roles of the majority of GPI-anchored proteins in filamentous fungi remain largely unknown, especially in *B. cinerea*.

CFEM (common in several fungal extracellular membrane proteins) domain is unique in fungi and contains eight conserved cysteine residues (Kulkarni et al., 2003; Zhang et al., 2015). It is similar to epidermal growth factor (EGF)-like domains which functions as extracellular receptor or signal transducers or as adhesion molecules in host–pathogen interactions (Appella et al., 1988; Kulkarni et al., 2003). The CFEM domain-containing proteins in *Candida albicans* and *C. glabrata* are involved in binding and maintenance of iron, adherence and virulence (Pérez et al., 2011; Sorgo et al., 2013; Srivastava et al., 2014). In plant pathogen fungus *Fusarium oxysporum*, the expressions of several CFEM_DR genes increased dramatically during colonizing and infecting on hosts (Ling et al., 2015). Further more, several reports suggested that the transcripts of CFEM domain-containing proteins have been detected in the transcriptome of many fungi (Pendleton et al., 2014; Vela-Corcía et al., 2016), especially in the secretome of broad-host-range necrotrophs *S. sclerotiorum* (Guyon et al., 2014). In *Magnaporthe oryzae*, the CFEM domain of seven-transmembrane protein Pth11, a significant G-protein-coupled receptor, is necessary for proper development of appressoria, appressoria-like structures and pathogenicity (Kou et al., 2017). However, the underlying mechanisms by which CFEM domain-containing proteins act remain largely unknown, especially in broad-host-range necrotrophic pathogen *B. cinerea*.

In this study, we isolated and characterized a CFEM protein BcCFEM1 (BC1G_15201) with a putative GPI modification site. Disruption of *BcCFEM1* gene results in decreased virulence and increased sensitivity to osmotic and cell wall stress, indicating that BcCFEM1 plays a key role in stress resistance and is required for virulence. Further more, transient expression of *BcCFEM1* gene by *Agrobacterium* infiltration in tobacco leaves triggers obvious chlorosis, suggesting its potential eliciting role in plant–pathogen interaction.

## Materials and Methods

### Fungal and Bacterial Strains, Plants, Growth Conditions, and Inoculation

*Botrytis cinerea* wild type (WT) strain B05.10 was used in this study. *B. cinerea* strains were routinely grown on potato dextrose agar (PDA, Difco) and maintained at 22°C under continuous fluorescent light. Conidia were obtained from 7-days-old cultures. The *Agrobacterium tumefaciens* strain GV3101 was used for *Agrobacterium*-mediated transient expression in plant leaves. *Escherichia coli* strain DH5α was used to propagate plasmids. Bean (*Phaseolus vulgaris* cv. French bean, genotype N9059) and tobacco (*Nicotiana benthamiana*) plants were grown in greenhouse (16: 8 h, 25: 22°C, day: night).

Pathogenicity assays of *B. cinerea* on beans were performed as described (Schumacher et al., 2012) that the conidia harvested from 7-days-old agar cultures were re-suspended in Gamborg’s B5 medium supplemented with 2% glucose and 10 mM KH_2_PO_4_/K_2_HPO_4_, pH 6.4. The primary leaves of 9-days-old beans were inoculated with 7.5 μl conidial suspensions (2 × 10^5^ conidia/ml) and the infected bean plants were incubated in the humid chambers at 22°C for 72 h, and the lesion diameter was measured.

### Bioinformatics Analysis in This Study

The genomic sequence database of *B. cinerea* at JGI^1^ was used to characterize *B. cinerea* genes. The *GPI Modification Site Prediction*^2^ was used to analyze potential GPI modification site. The *SMART MODE*^3^ was used to analyze signal peptide sequence and protein domain. Database *NCBI* was used for Blastp analysis. The *Clustal W* and *Jalview* program were used for mature proteins alignments. *MEGA 5* program was used to generate phylogenetic tree with unrooted neighbor-joining method.

### Extraction and Manipulation of DNA and RNA

To explore the expression pattern of *BcCFEM1* during early infection stage, the *B. cinerea* conidial suspensions (5 × 10^5^ conidia/ml) were sprayed onto the beans leaves (9-days-old). The inoculated leaves and the hyphae growing on plates (solid Gamborg’s B5 medium containing 2% glucose) as control were harvested at 12, 24, 36, 48, 60, and 72 hours post inoculation (hpi), respectively, and then frozen at -80°C for total RNA extraction. To compare expression level of *BcCFEM1* between WT strain, *BcCFEM1* deletion mutant and *BcCFEM1* over-expression strain, the mycelial agar disks of these strains were inoculated to the cellophane of PDA at 22°C for 3 days, and mycelia were then collected and stored at -80°C for total RNA extraction.

The genomic DNA of fungi were isolated with Axygen DNA kit (Axygen, United States) according to the manufacturer’s protocols. The total RNA samples of fungal strains and plants were isolated using the Axygen RNA kit (Axygen, United States) according to the manufacturer’s instructions and stored at -80°C for further study. The DNase I (TAKARA Biotechnology, Dalian, China) treatment was performed to eliminate residual genomic DNA and the RevertAid^TM^ First Strand cDNA Synthesis Kit (Thermo Scientific, Lithuania) was used to generate the first strand cDNA.

Quantitative RT-PCR (qRT-PCR) was performed by the using of CFX96 Touch^TM^ Real-Time PCR Detection System (Bio-Rad, Hercules, CA, United States) and SYBR^®^Premix Ex Taq^TM^ II (TAKARA Biotechnology, Dalian, China), according to the manufacturer’s instructions. Primers were designed across or flanking an intron (Supplementary Table S1). The relative expression of *B. cinerea Bcgpdh* (*BC1G_05277*) gene (Ma et al., 2017) and *P. vulgaris Actin-11* (GenBank: EH040443.1) gene (Oliveira et al., 2015) were respectively used as reference genes for normalizing the RNA sample. For each gene, qRT-PCR assays were respectively repeated at least twice, with each repetition having three independent replicates.

Southern blot analysis was performed following the method described (Wei et al., 2016) that 15–20 μg of genomic DNA of each strain was digested with *Xba*I, size-fractionated through a 0.8% agarose gel and mounted on positively charged nylon membrane. The nylon membrane was then hybridized with a probe amplified from hygromycin *hph* gene by primer pairs (Supplementary Table S1) and labeled with digoxigenin (DIG)-dUTP using the PCR DIG Probe Synthesis Kit (Roche, Mannheim, Germany) following the manufacturer’s protocols.

### Construction of Plasmids and Transformation of *B. cinerea*

The *BcCFEM1* replacement constructs was generated as described (Ma et al., 2017) that the 5′(547 bp)- and 3′(536 bp)- flanks of the *BcCFEM1* ORF were amplified from genomic DNA of the WT strain B05.10 with primer pairs (Supplementary Table S1). The fragments were then respectively cloned into the upstream and downstream of *hph* cassette using Gibson Assembly Master Mix kit (New England Biolabs, Ipswich, MA, United States) according to the manufacturer’s protocols.

For complementation assays, the PCR product containing a 2-kb upstream sequence, a full-length *BcCFEM1* gene coding region and a 1-kb downstream sequence was amplified from the genomic DNA of WT strain using primer pairs (Supplementary Table S1), and cloned into the plasmid p3300neoIII to generate the complementary vector p3300neoIIIBcCFEM1 as described (Zhang et al., 2016).

To construct the over-expression vector of *BcCFEM1*, the full-length *BcCFEM1* ORF fused with a HA tag at C terminus was cloned into *hph* cassette-containing vector p3300hyg under the manipulation of *PtrpC* promoter and *TtrpC* terminator as described (Wei et al., 2016). To construct the expression vector of *SP-EGFP-HA*, *EGFP* ORF fused with BcCFEM1 signal peptide and HA tag at N and C terminus respectively was cloned into *hph* cassette-containing vector p3300hyg under the manipulation of *PtrpC* promoter and *TtrpC* terminator.

For transient expression in *N. benthamiana*, the sequence encoding *Arabidopsis thaliana* PR3 (*TAIR* ID: AT3G12500) signal peptide-BcCFEM1-HA tag fusion protein was cloned into pCAMBIA3300 under the manipulation of CaMV 35S promoter and NOS terminator. The transient expressions of other genes were also performed using this method.

Protoplasts preparation and genetic transformation of *B. cinerea* were performed as previously described (Ma et al., 2017).

### Characterization of *B. cinerea* Transformants

To assay growth rate, the WT strain, *BcCFEM1* deletion mutants, and *BcCFEM1-HA* over-expression transformants were cultured on PDA at 22°C for 2 days. Then, the mycelial agar disks were taken from the active colony edge and inoculated on the center of the PDA petri dish at 22°C to examine the hyphal growth rate. The colony morphology of these strains were examined after being grown on PDA plate for 7 days at 22°C under 24 h fluorescent light photoperiod and/or 14 days at 22°C under dark condition. For stress tolerance assay, indicated strains were respectively inoculated onto PDA plates containing 1 M NaCl, 1 M Sorbitol, 0.4 mg/ml Calcofluor White (CFW), 20 mM H_2_O_2_, 0.8 mg/ml Congo Red (CR) and 0.02% SDS as previously described (Ma et al., 2017). To analyze the conidial germination, 25 μl conidial suspensions (1 × 10^5^ conidia/ml) of each strain was spotted on hydrophobic coverslips and incubated at 22°C for 10 h. Then, the conidial germination was examined by Nikon Eclipse 80i microscope (Nikon, Tokyo, Japan), under bright-field model using 40× fold magnification.

### Transient Expression, Protein Extractions, and Immunoblot Analysis

*Agrobacterium*-mediated transient expression was performed using leaf infiltration as described (Kettles et al., 2017). Protein extractions from *B. cinerea* and the immunoblot analysis were performed as described (Lyu et al., 2016).

To confirm whether the *BcCFEM1*-HA fusion protein was secreted outside the cell to extracellular matrix, 0.2 g fresh mycelia was cultured in 5 ml potato dextrose broth (PDB, Difco) in 6-well-cell culture plate at 22°C with agitation at 180 rpm for 3 days. The liquid mediums were then filtered with 0.45 μm Minisart^®^non-pyrogenic filter (Sartorius-Stedim Biotech, Germany) to eliminate residual mycelia. The Amicon^®^Ultra-4 Centrifugal Filter Devices (10 ml, 10 kDa, Merck Millipore, Billerica, MA, United States) was used to clean and concentrate the target secretory protein solution. The clean samples were dissolved in 250 μl PBS solution and frozen at -80°C for further immunoblot analysis.

### Statistical Analysis

Statistical tests were performed using Origin 7.5 (OriginLab Corporation, Northampton, MA, United States). Data significance was analyzed by the using of ANOVA (one-way, *P* ≤ 0.01). In all graphs, results represent the mean value of three independent experiments ± SD. Different letters or asterisks in the graphs indicate statistical differences, *P* ≤ 0.01.

## Results

### BC1G_15201 Has a Signal Peptide and Contains a CFEM Domain

**FIGURE 1:**
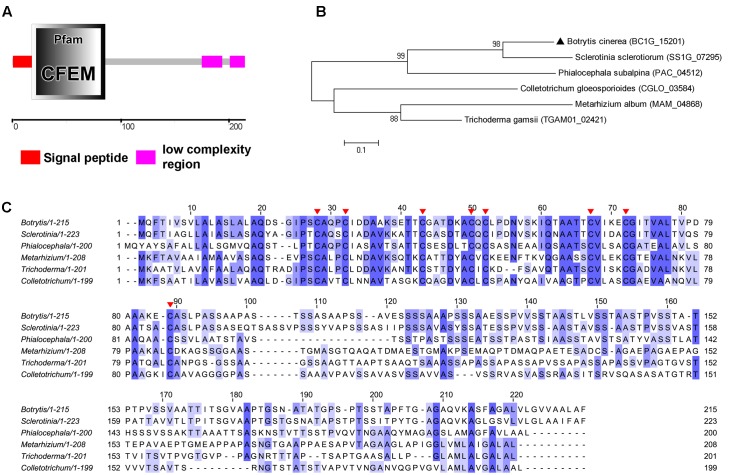
Sequence similarities between BcCFEM1 and other CFEM proteins. **(A)** Schematic illustrations of BcCFEM1. The domain of BcCFEM1 was predicted by *SMART* web site. **(B)** Phylogenetic analysis of BcCFEM1 and CFEM proteins from other fungi. The full length protein sequences were analyzed by *MEGA 5* with Unrooted neighbor-joining bootstrap (1,000 replicates). The black triangle indicates BcCFEM1. A scale bar at the lower left corresponds to a genetic distance of 0.1. **(C)** Multiple sequence alignment of BcCFEM1 and other CFEM proteins. Full-length of protein sequences were aligned using *Clustal W* and the alignment was edited using *Jalview*. Eight conserved cysteine residues are highlighted with red triangles. Blue shading intensity reflects level of amino acid identity at each position. *Botrytis*: BcCFEM1 (BC1G_15201); *Sclerotinia*: SS1G_07295; *Metarhizium*: MAM_04868; *Phialocephala*: PAC_04512; *Colletotrichum*: CGLO_03584; *Trichoderma*: TGAM01_02421.

The *B. cinerea BC1G_15201* gene is a single copy gene, consisting of two exons, encoding 215 amino acid. The *SMART MODE* analysis result indicated that the initial N terminus 17 amino acids encode signal peptide and 18–86 amino acids encode CFEM (common in several fungal extracellular membrane proteins) domain (Pfam ID: PF05730) (**Figure 1A**). No transmembrane helices of this protein were predicted, indicating that BC1G_15201 is a secretory protein possibly. BLAST searches for homologous sequences of BC1G_15201 only resulted in similarity with sequences from *Sclerotinia sclerotiorum* (SS1G_07295, E-value: 2E-52, 58% identity), *Metarhizium album* (MAM_04868, E-value: 2E-15, 36% identity), *Phialocephala subalpina* (PAC_04512, E-value: 2E-13, 37% identity), *Colletotrichum gloeosporioides* (CGLO_03584, E-value: 2E-09, 45% identity), *Trichoderma gamsii* (TGAM01_02421, E-value: 6E-09, 58% identity) that match to CFEM domain. Phylogenetic and multiple sequence alignment analysis revealed sequences conservation (**Figures 1B,C**), especially the eight cysteine residues (C^25^, C^29^, C^40^, C^47^, C^49^, C^64^, C^69^, and C^85^) in BC1G_15201 are well-conserved in all of these homologs (**Figure 1C**), which may be involved in the formation of disulfide bonds and play significant roles in the structure and function of BC1G_15201.

Therefore, we named BC1G_15201 protein “BcCFEM1,” as this is the first report of *B. cinerea* CFEM protein.

### BcCFEM1 Contains a Putative GPI-Anchored Site

*GPI Modification Site Prediction*^4^ was used to predict potential GPI modification site. The result demonstrated that the amino acids G^192^ and A^193^ are predicted to be the best and second best of potential GPI-modification site, respectively (**Supplementary Figure S1**). From this result, we inferred that BcCFEM1 contains a putative GPI-anchored site, which possibly anchored to the outer layer of the plasma membrane through a C-terminal GPI anchor or transferred to the cell wall as other fungal GPI-anchored proteins (Krüger et al., 2016).

### BcCFEM1 Contains a Signal Peptide

**FIGURE 2:**
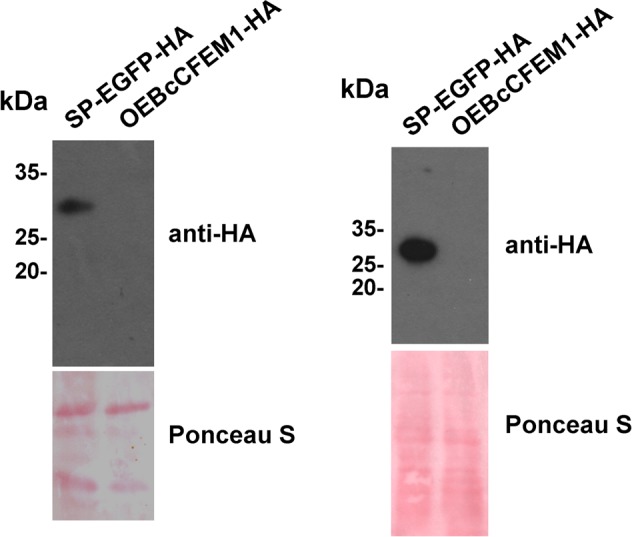
BcCFEM1-HA is not detected in mycelia. Immunoblot analysis with secretory proteins (left panel) and total proteins of mycelia (right panel) isolated from 3-days-liquid PDB culture of *Botrytis cinerea* transformants respectively over expressing BcCFEM1-HA and SP-EGFP-HA. SP-EGFP-HA and BcCFEM1-HA proteins were detected using anti-HA antibody. Ponceau S staining of total secretory proteins indicate equal protein loading.

As mentioned above that BcCFEM1 contains a putative signal peptide and maybe a secretory protein, to verify this hypothesis further, SP-EGFP-HA expression strain, which EGFP fused with BcCFEM1 signal peptide and HA tag at N and C terminus respectively, was constructed and cultured in PDB medium for shake culture. Western blot result demonstrated that SP-EGFP-HA fusion protein could be detected in both culture medium and mycelium (**Figure 2**). The result indicated that the signal peptide from BcCFEM1 can mediate secretion of protein effectively.

In addition, we also constructed over expression strain OEBcCFEM1-HA and cultured in PDB medium. Unexpectedly, Western blot result demonstrated that BcCFEM1-HA fusion protein could not be detected in both mycelium and culture medium (**Figure 2**), indicating that BcCFEM1-HA fusion protein may be cleaved or modified.

### *BcCFEM1* Expresses Highly at the Early Stages of Infection and Induces Chlorosis in *N. benthamiana* Leaves

**FIGURE 3:**
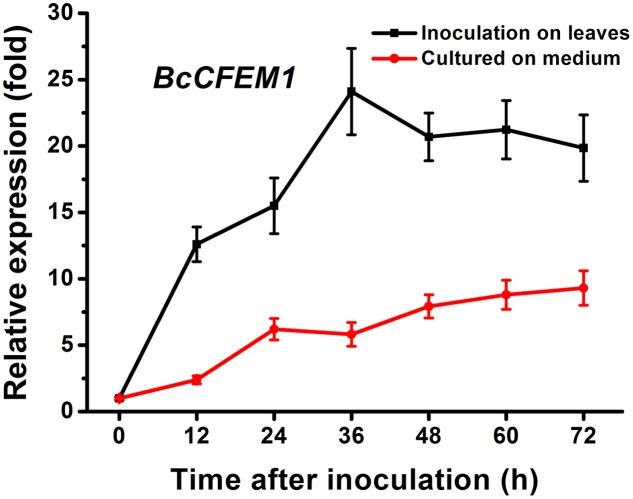
The relative expression pattern of *BcCFEM1* gene detected with qRT-PCR after growing on Gamborg’s B5 medium (red line) or inoculating on bean plants (dark line) for 0–72 h. The relative expression was calculated by the comparative Ct method. The *BcCFEM1* expression level of *B. cinerea* inoculated on Gamborg’s B5 medium at 0 hpi was set as level 1. The transcript level of *B. cinerea Bcgpdh* gene (Ma et al., 2017) was used to normalize different samples. Data represent means and standard deviations (three independent replicates).

To explore the possible contribution of BcCFEM1 to *B. cinerea*, we determined the expression patterns of *BcCFEM1* during infection stages following inoculation on beans with conidia. Results indicated that when *B. cinerea* was inoculated on bean leaves, the transcript levels of *BcCFEM1* increased from 12 to 36 hpi, and then decreased, but the expression level was still higher than at 0 hpi (**Figure 3**). However, when inoculated on solid Gamborg’s B5 medium, the expression of *BcCFEM1* did not vary to any great extent, with the highest expression being observed at late stage (72 hpi) (**Figure 3**). Therefore, the expression levels of *BcCFEM1* must be induced by its interaction with host plants, suggesting that this gene may play significant roles at the early stages of infection.

**FIGURE 4:**
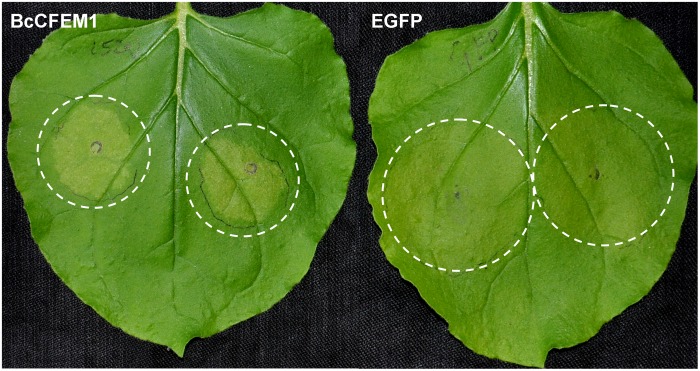
BcCFEM1 induces chlorosis in *Nicotiana benthamiana*. Images of *N. benthamiana* leaves 5 days after agroinfiltration using constructs encoding BcCFEM1-HA and SP-EGFP-HA, respectively.

In addition, transient expression of *BcCFEM1-HA* using *Agrobacterium*-infiltration method caused obvious chlorosis in *N. benthamiana* leaves (**Figure 4**), indicating that BcCFEM1 may function as a virulence factor.

### BcCFEM1 Is not Required for Growth, Conidial Germination, and Colony Morphology

We firstly constructed *BcCFEM1* deletion, complementary and over-expression strains, and these strains were confirmed by PCR, RT-PCR, qRT-PCR, and Southern blot (**Supplementary Figure S2**).

**FIGURE 5:**
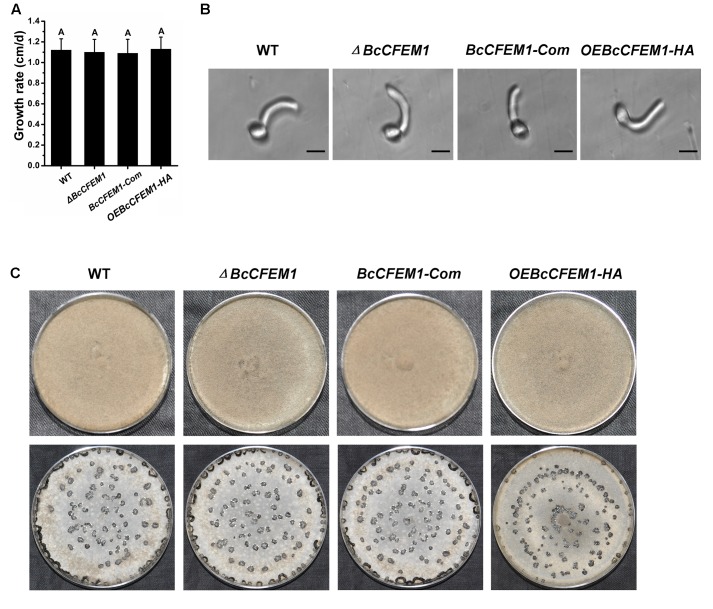
Deletion or over expression of *BcCFEM1* does not affect growth rate, conidial germination, sclerotial formation, and colony phenotype. **(A)** Hyphal growth rates of *BcCFEM1* deletion mutant and over-expression transformant. Growth rates were examined on PDA at 22°C. Data represent means and SD from three independent experiments, each with three replications. The same letters in the graph indicate no statistical differences at *P* ≤ 0.01 using one-way ANOVA. **(B)** Images of conidia of each strain spotted on hydrophobic coverslips and incubated at 22°C for 10 h. The conidial germination was examined by Nikon Eclipse 80i microscope (Nikon, Tokyo, Japan), under bright-field model using 40× fold magnification. Bars = 10 μm. **(C)** Colony morphology of *BcCFEM1* deletion mutant and over-expression transformant. Upper panel, colonies were grown on PDA at 22°C for 7 days under 24 h light photoperiod; lower panel, colonies were grown on PDA at 22°C for 15 days under dark condition.

To establish whether *BcCFEM1* is required for *B. cinerea* physiological processes, the phenotype of WT, *BcXYG1* deletion mutant *ΔBcCFEM1*, *BcCFEM1* complementary strain *BcCFEM1-*Com and *BcCFEM1* over-expression strain *OEBcCFEM1* were assayed. The result showed that there was not any obvious difference in growth rate between these strains when cultured on PDA medium (**Figure 5A**). In addition, the conidial morphology and germination were also assayed. However, we did not detect any differences in spore germination between the WT, *ΔBcCFEM1*, *BcCFEM1-*Com, and *OEBcCFEM1* strains (**Figure 5B**). Further more, the colony morphology of each strain under continuous fluorescent light or dark condition was also analyzed. Likewise, over expression or deletion of *BcCFEM1* gene had no visible effect on colony morphology and sclerotial production on PDA plate (**Figure 5C**).

### Deletion of *BcCFEM1* Affects Fungal Virulence, Conidiation, and Stress Tolerance

**FIGURE 6:**
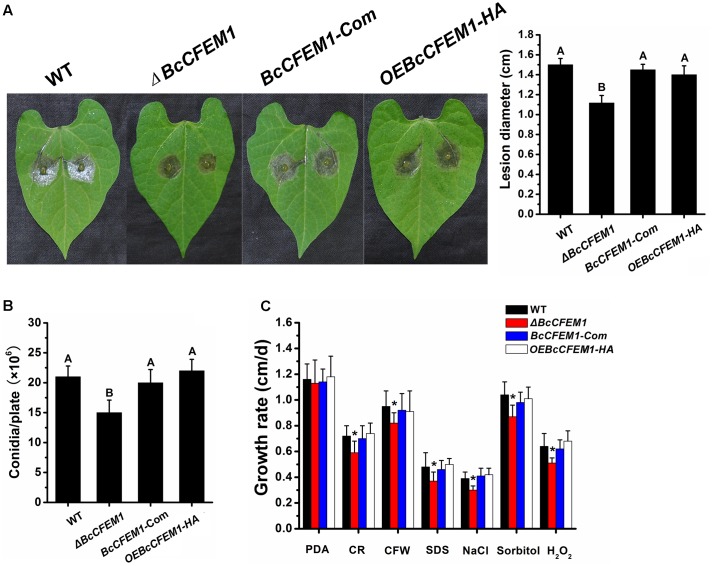
Deletion of *BcCFEM1* affects pathogenicity, conidial production, and stress tolerance. **(A)** Virulence analysis of indicated strains. The beans leaves were inoculated with 7.5 μl conidial suspensions (2 × 10^5^ conidia/ml) and incubated in the humid chambers at 22°C for 72 h, and then the lesions were measured. Data represent means and SD from three independent experiments, each with six replications. Different letters in the graph indicate statistical differences at *P* ≤ 0.01 using one-way ANOVA. **(B)** Conidial production of indicated strains. strains were grown on PDA at 22°C for 7 days under 24 h light photoperiod. Data represent means and SD from three independent experiments, each with three replications. Different letters in the graph indicate statistical differences at *P* ≤ 0.01 using one-way ANOVA. **(C)** Hyphal growth rates of the indicated strains in PDA medium containing 1 M NaCl, 1 M Sorbitol, 0.3 mg/ml Calcofluor White (CFW), 0.5 mg/ml Congo Red (CR), 20 mM H_2_O_2_ and 0.02% SDS, respectively. Growth rates were examined on PDA at 22°C. Data represent means and SD from three independent experiments, each with three replications. Asterisks indicate statistical differences between *BcCFEM1* deletion mutant and other strains in a given medium at *P* ≤ 0.01 using one-way ANOVA.

As described above that the *BcCFEM1* was induced to express highly at early infection stage and could cause chlorosis in tobacco leaves, inferring that BcCFEM1 may be involved in fungal virulence. To study the possible effect of *BcCFEM1* on *B. cinerea* virulence, infection was carried out on bean leaves. Results demonstrated that the pathogenicity of *ΔBcCFEM1* was decreased compared to WT, *BcCFEM1-*Com and *OEBcCFEM1* strains, but over expression of *BcCFEM1* had no visible effect on virulence (**Figure 6A**).

Although WT, *ΔBcCFEM1*, *BcCFEM1-*Com, and *OEBcCFEM1* cultures developed smooth and uniform mycelium that differentiated to form abundant spores within 7 days, conidial production of *ΔBcCFEM1* was reduced compared with WT, *BcCFEM1-*Com and *OEBcCFEM1* strains (**Figure 6B**).

In addition, to investigate whether BcCFEM1 is involved in external stress tolerance, growth assays of indicated strains on PDA media supplemented with salt stress (1 M NaCl), osmotic stress (1 M Sorbitol), H_2_O_2_ (20 mM) and cell wall stress (0.3 mg/ml CFW, 0.02% SDS, and 0.5 mg/ml CR) were respectively analyzed as previously described (Pérez-Hernández et al., 2017). The results showed that the *ΔBcCFEM1* strain was more hypersensitive to all of these compounds compared with WT, *BcCFEM1-*Com and *OEBcCFEM1* strains (**Figure 6C**). However, no obvious change in stress tolerance between WT, *BcCFEM1-*Com and *OEBcCFEM1* strain was detected (**Figure 6C**). These results indicated that BcCFEM1 may positively regulate the sensibility of *B. cinerea* to cell wall stressors, oxidative stress and osmotic stabilizers.

## Discussion

In this study, we identified a CFEM domain-containing protein BcCFEM1 with putative GPI-anchored site in *B. cinerea*. CFEM domain have been reported to function as cell surface transmembrane receptor or adhesion molecule during host–pathogen interactions (Pérez et al., 2006; Pérez et al., 2011; Kou et al., 2017). Recent study identified a novel CFEM G-protein coupled receptor, WISH, which is indispensable for surface sensing, asexual and pathogenic differentiation in *M. oryzae* (Sabnam and Barman, 2017). Other studies have also screened several CFEM domain-containing proteins by analysis of transcriptome during infection stage (Guyon et al., 2014; Vela-Corcía et al., 2016; Seifbarghi et al., 2017), suggest their potential pathogenicity functions. Indeed, in our study, deletion of *BcCFEM1* resulted in decreased virulence on bean leaves (**Figure 6A**), indicated that BcCFEM1 functions as a virulence factor.

Beside transmembrane CFEM proteins, some putative secreted proteins containing CFEM domains have also been screened as candidate secreted effectors in several pathogens by transcriptome analysis (Vela-Corcía et al., 2016; Seifbarghi et al., 2017). In our study, transient expression of BcCFEM1 fused with signal peptide which targeted to the apoplast induced obvious chlorosis in tobacco leaves (**Figure 4**), indicating that BcCFEM1 may function as an apoplastic elicitor that targeted to the extracellular space of *N. benthamiana* cells to induce chlorosis. Similar findings were also reported in recent study that secretion of several apoplastic effectors from *Zymoseptoria tritici* are required to induce plant cell chlorosis or death (Kettles et al., 2017). However, the interact protein of BcCFEM1 and the chlorosis-inducing signal pathway are still unknown, and require further study.

As BcCFEM1 was predicted to contain a signal peptide but no transmembrane structure (**Figure 1A**), indicating that BcCFEM1 may be a potential secreted protein. In our study, EGFP fused with BcCFEM1 signal peptide and HA tag at N and C terminus respectively could be detected in both culture medium and mycelium of SP-EGFP-HA expression transformant (**Figure 2**), indicating that the signal peptide from BcCFEM1 can mediate secretion of protein effectively. Furthermore, we have also tried several times to analyze whether BcCFEM1-HA fusion protein also secrete into culture medium using Western blot. However, BcCFEM1-HA fusion protein could not be detected in both mycelium and culture medium of *BcCFEM1-HA* over expression transformant (**Figure 2**). In addition, we also expressed *BcCFEM1-HA* in *ΔBcCFEM1* mutant and the deficiency of *ΔBcCFEM1* mutant in pathogenicity, conidiation and stress tolerance could be rescued (**Supplementary Figure S3**). Thus, these results indicated that BcCFEM1-HA fusion protein functions and may be cleaved or modified but not degraded when expressed in *B. cinerea*. However, the underlying mechanism still need to be further analyzed.

Then, we also analyzed the potential modification of BcCFEM1. The result demonstrated that G^192^ and A^193^ are predicted to be the potential GPI-modification site (**Supplementary Figure S1**), therefore, BcCFEM1 may be a potential GPI-anchored CFEM protein, which anchored to the outer layer of the cell membrane through a C-terminal GPI anchor or transferred to the cell wall possibly (Krüger et al., 2016). Until now, several studies have reported that GPI-anchored proteins anchor to the plasma membrane and play an important role in maintaining the cell wall and stress tolerance. In *Candida* species, CFEM proteins were involved in biofilm formation and iron acquisition (Ding et al., 2011). In plants, GPI-anchored proteins involved in regulation of cell expansion and cell wall biosynthesis (Brady et al., 2007). In *S. cerevisiae*, several GPI-modified wall proteins are important to osmotic stability and mortality (Teparić et al., 2004). GPI-anchored membrane proteins DCW1 and DFG5 are essential for cell wall biogenesis in *S. cerevisiae*, double deletion caused fragile cell wall and is lethal (Kitagaki et al., 2002, 2004). In *M. oryzae*, the CFEM protein Pth11 is a transmembrane protein and functions as G-protein-coupled receptor (Kou et al., 2017). However, to identify whether BcCFEM1 is a GPI-anchored protein or a secreted elicitor protein, further studies need to be explored.

In our study, deletion of *BcCFEM1* caused obvious higher sensitive to cell wall and stress tolerance (**Figure 6C**), indicating that BcCFEM1 positively regulate the tolerance of *B. cinerea* to cell wall stressors and osmotic stabilizers. In addition, previous study reported that the cysteines in CFEM of *M. oryzae* possess antioxidant properties (Kou et al., 2017). Similar result is also observed in our study that deletion of *BcCFEM1* in *B. cinerea* results in enhanced sensitivity to H_2_O_2_ (**Figure 6C**). Thus, we inferred that the reduced virulence of deletion mutant may be the result of its high sensitivity to defense-associated reactive oxygen species (ROS) of plants. However, there was not obvious difference in virulence, conidiation and stress tolerance between *BcCFEM1-HA* over expression strain and the WT strain (**Figure 6**). Similar result had also been reported in other studies that over expression of virulence-related protein or elicitor protein do not affect the virulence and other phenotypes (Frías et al., 2016; Lyu et al., 2016). In our study, the rapid increase of *BcCFEM1* expression level in WT strain during infection stage (**Figure 3**) could possibly explain the lack of difference in virulence between WT strain and *BcCFEM1-HA* over expression strain.

In summary, we have identified a CFEM domain-containing protein BcCFEM1 with putative GPI-anchored site involved in virulence, conidiation and stress tolerance. In addition, BcCFEM1 could also induce chlorosis in *N. benthamiana* leaves. Given these properties, targeting CFEM proteins could be a good strategy to control gray mold diseases. The results described above will also enhance our understanding of the interaction mechanisms between *B. cinerea* and plants. However, our finding also arises more important questions to be answered, such as, is BcCFEM1 a GPI-anchored protein or a secreted elicitor protein? Which epitopes of BcCFEM1 contribute to its chlorosis-inducing activity? Which proteins recognize and interact with BcCFEM1? Which pathways transmit the chlorosis-inducing and stress tolerance signaling? We will explore these issues in future studies.

## Conclusion

The CFEM domain-containing protein BcCFEM1 with putative GPI-anchored site was experimentally confirmed to be involved in pathogenicity, conidial production and stress tolerance in *B. cinerea*. Furthermore, our results also firstly showed that transient expression of *BcCFEM1* in *N. benthamiana* caused obvious chlorosis, indicating that BcCFEM1 plays diverse roles in this fungus.

## Author Contributions

Conceived and designed the experiments: WZ and WW. Performed the experiments: WZ, WW, YW, and YZ. Analyzed the experiment data: WZ, WW, YW, YZ, FP, SZ, PC, and XX. Contributed reagents/materials/analysis tools: WZ, FP, SZ, and PC. Wrote the paper: WZ and WW. All authors have read and approve the final manuscript.

## Conflict of Interest Statement

The authors declare that the research was conducted in the absence of any commercial or financial relationships that could be construed as a potential conflict of interest.

## Footnotes

1 http://genome.jgi.doe.gov/Botci1/Botci1.home.html

2 http://mendel.imp.ac.at/gpi/plant_server.html

3 http://smart.embl-heidelberg.de/smart/change_mode.pl

4 http://mendel.imp.ac.at/gpi/plant_server.html

## Supplementary Material

**FIGURE S1 |** GPI site prediction of BcCFEM1. The *GPI Modification Site Prediction* (http://mendel.imp.ac.at/gpi/plant_server.html) was used to analyze potential GPI modification site of BcCFEM1.

**FIGURE S2 |** The *BcCFEM1* gene deletion strategy and confirmation. **(A)** Strategy used to generate the *BcCFEM1* deletion mutant *ΔBcCFEM1*. The deletion construct used to transform the wild type strain contain the hygromycin resistance (*hph*) cassette flanked by upstream and downstream sequence of *BcCFEM1* gene. The positions of the PCR primers used to verify the deletion transformants are indicated (*BcCFEM1* Rep-up F and *BcCFEM1* Rep-down R). The scale bar indicates 500 bp. **(B)** Southern blot analysis. Genomic DNA (15 μg per lane) of indicated strains was digested with *Xba*I. The same filter was hybridized with a probe corresponding to the *hph* cassette (P). **(C)** PCR amplifications carried out to check the *BcCFEM1* deletion mutants using the primers *BcCFEM1* Rep-up F and *BcCFEM1* Rep-down R, and the template: genomic DNA from indicated strains. **(D)** Analysis of *BcXYG1* expression in indicated strains with RT-PCR. The *B. cinerea Bcgpdh* gene was used to normalize different samples. **(E)** qRT-PCR carried out to analyze the expression levels of *BcCFEM1* gene in indicated strains. The relative levels of transcript were calculated by the comparative Ct method. The *BcCFEM1* gene expression level in WT was set as level 1. The transcript level of *B. cinerea Bcgpdh* gene was used to normalize different samples. Data represent means and standard deviations (three independent replicates). ND, not detected.

**FIGURE S3 |** Expression of *BcCFEM1-HA* in *ΔBcCFEM1* mutant (*ΔBcCFEM1-C*) could rescue the deficiency of *ΔBcCFEM1* mutant in pathogenicity, conidiation, and stress tolerance. **(A)** Virulence analysis of indicated strains. The beans leaves were inoculated with 7.5 μl conidial suspensions (2 × 10^5^ conidia/ml) and incubated in the humid chambers at 22°C for 72 h, and then the lesions were measured. Data represent means and SD from three independent experiments, each with six replications. Different letters in the graph indicate statistical differences at *P* ≤ 0.01 using one-way ANOVA. **(B)** Conidial production of indicated strains. strains were grown on PDA at 22°C for 7 days under 24 h light photoperiod. Data represent means and SD from three independent experiments, each with three replications. Different letters in the graph indicate statistical differences at *P* ≤ 0.01 using one-way ANOVA. **(C)** Hyphal growth rates of the indicated strains in PDA medium containing 1 M NaCl, 1 M Sorbitol, 0.3 mg/ml Calcofluor White (CFW), 0.5 mg/ml Congo Red (CR), 20 mM H_2_O_2_ and 0.02% SDS, respectively. Growth rates were examined on PDA at 22°C. Data represent means and SD from three independent experiments, each with three replications. Asterisks indicate statistical differences between *BcCFEM1* deletion mutant and other strains in a given medium at *P* ≤ 0.01 using one-way ANOVA.
