# Supplementary figures and images for: BcCFEM1, a CFEM Domain-Containing Protein with Putative GPI-Anchored Site, Is Involved in Pathogenicity, Conidial Production, and Stress Tolerance in Botrytis cinerea
Source: Front Microbiol. 2017 Sep 20;8:1807. doi: 10.3389/fmicb.2017.01807 (PMC5611420; doi:10.3389/fmicb.2017.01807)

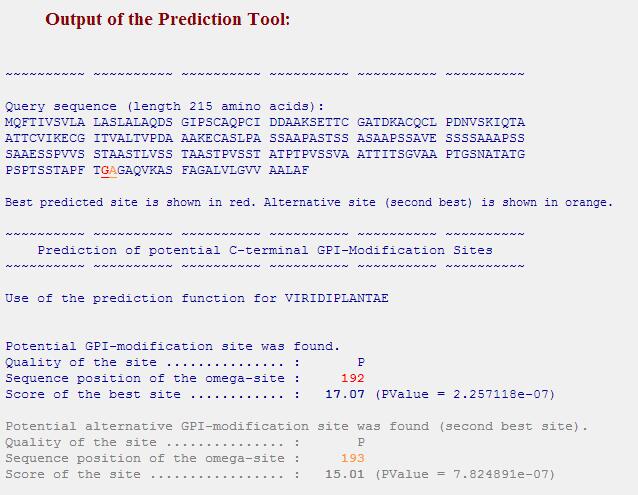

Supplement: Supplementary file 1 [file Image_1.JPEG]

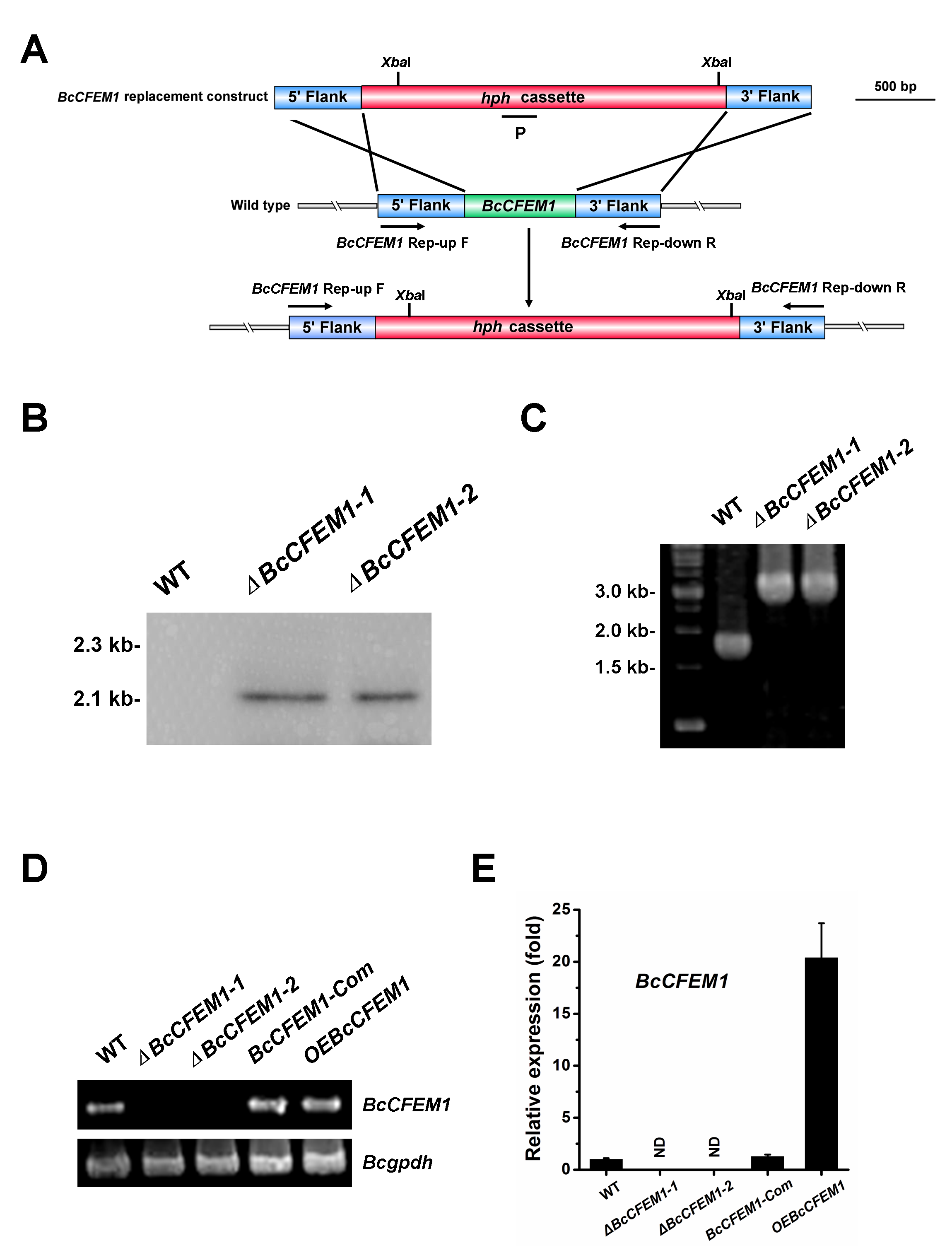

Supplement: Supplementary file 2 [file Image_2.TIF]

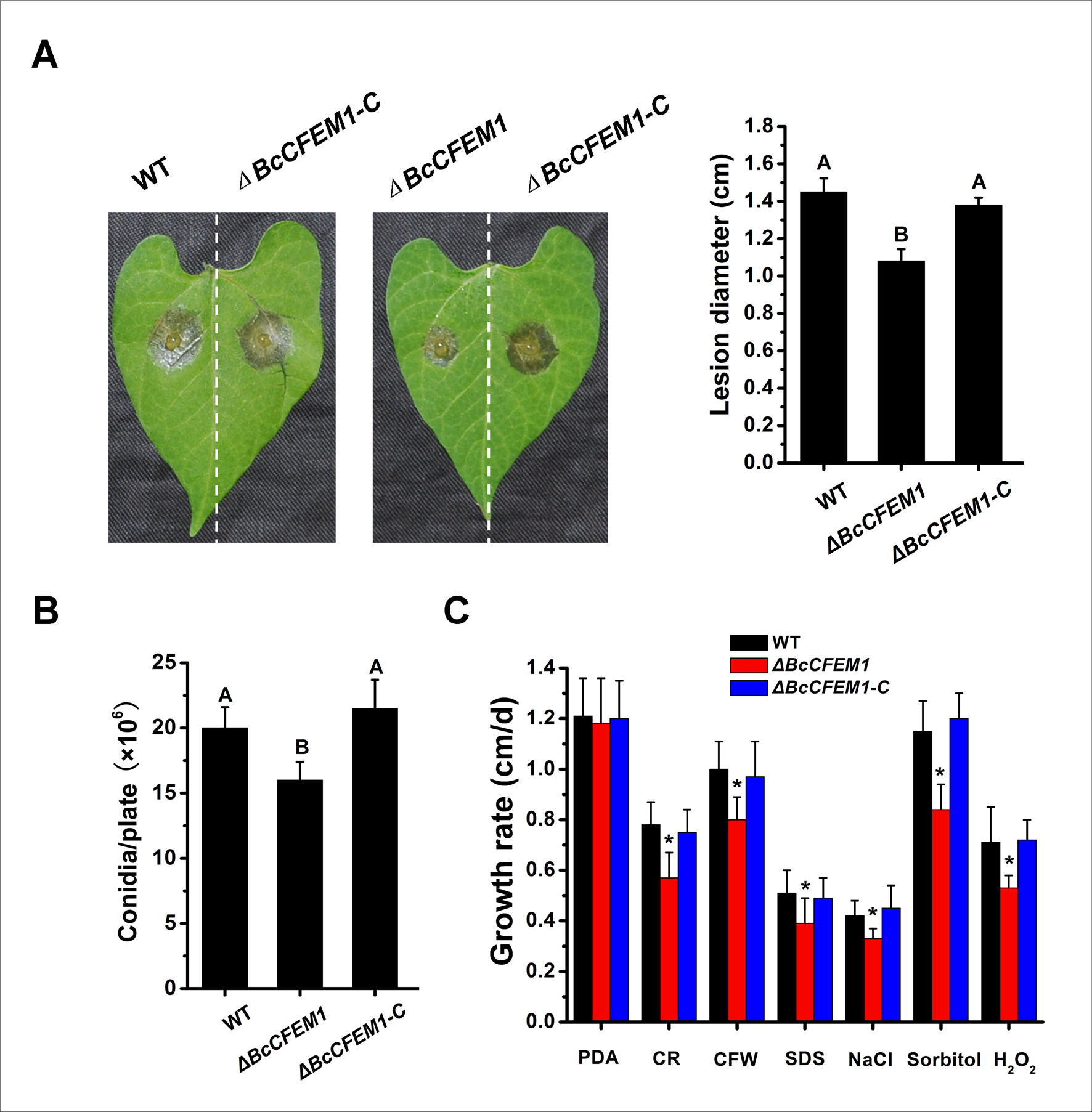

Supplement: Supplementary file 3 [file Image_3.TIF]
