# Supplementary material for: BcCFEM1, a CFEM Domain-Containing Protein with Putative GPI-Anchored Site, Is Involved in Pathogenicity, Conidial Production, and Stress Tolerance in Botrytis cinerea
Source: Front Microbiol. 2017 Sep 20;8:1807. doi: 10.3389/fmicb.2017.01807 (PMC5611420; doi:10.3389/fmicb.2017.01807)
Supplement: Supplementary file 4 [file Table_1.DOCX]

**Table S1. Primers used for vector construction and PCR.**

| **Primer purpose Primer name and sequence** | |
| --- | --- |
| *BcCFEM1* deletion for upstream | *BcCFEM1* Rep-up F: 5’ TAATGTTGTTCCTGCATCACAATG 3’  *BcCFEM1* Rep-up R: 5’CCTTTTTTTTTCAGGAATTATTCTCACAGTGATGGTTATTTGAGTATAAGGATTG 3’ |
| *BcCFEM1* deletion for downstream | *BcCFEM1* Rep-down F: 5’GATCTAGATGCATTCGCGAGGTACCGAGCTGCAATCGGACGAATGATAAGGATAT 3’  *BcCFEM1* Rep-down R: 5’ GTATCACCGAAGCAAGCATCGAGG 3’ |
| BcCFEM1 complementation | *BcCFEM1-*Com F: 5’ CGAGGTGGACGAAGGTTAGACAGAT 3’  *BcCFEM1-*Com R: 5’ GACAGAACGAATTCGTTTCTTTCGA 3’ |
| *BcCFEM1* over expression | *BcCFEM1-*OE F: 5’ AGGCGCGCCATGCAATTCACCATCGTCTCAG 3’  *BcCFEM1-*OE R: 5’ TATGCGGCCGCTTAAGCGTAATCTGGAACATCGTATGGGTAGAAGGCAAGG GCAGCGACGAC 3’ |
| *BcCFEM1* transient expression | *BcCFEM1-*TE R: 5’ AGGCGCGCCCCCAAGACAGCGGAATTCCAAGTTG 3’  *BcCFEM1-*TE R: 5’ TATGCGGCCGCTTAAGCGTAATCTGGAACATCGTATGGGTAGAAGGCAAGG GCAGCGACGAC 3’ |
| Probe of *hph* gene | Probe F: 5’ TCGGTCAATACACTACATGGCGTGA 3’  Probe R: 5’ GCCTCCAGAAGAAGATGTTGGCGAC 3’ |
| *BcCFEM1* for qRT-PCR | *BcCFEM1*-q F: 5’ AGGAGTGTGGTATCACCGTCGCTTT 3’  *BcCFEM1*-q R: 5’ AGCAGAAGATGGAGCAGCGCTAG 3’ |
| *B. cinerea Bcgpdh* for qRT-PCR | *Bcgpdh*-q F: 5’CGAAGAATAGCACAAACAGCTGGAC 3’  *Bcgpdh*-q R: 5’CGTCACCTTATGCTTCTTGCTCC 3’ |
